# Growth-Enhanced Transgenic Coho Salmon (*Oncorhynchus kisutch*) Strains Have Varied Success in Simulated Streams: Implications for Risk Assessment

**DOI:** 10.1371/journal.pone.0169991

**Published:** 2017-01-09

**Authors:** Rosalind A. Leggatt, L. Fredrik Sundström, Krista Woodward, Robert H. Devlin

**Affiliations:** Centre for Aquaculture and Environmental Research, Centre for Aquatic Biotechnology Regulatory Research, Fisheries and Oceans Canada, West Vancouver, British Columbia, Canada; Northwest Fisheries Science Center, UNITED STATES

## Abstract

Growth hormone (GH) transgenic fish have accelerated growth and could improve production efficiency in aquaculture. However, concern exists regarding potential environmental risks of GH transgenic fish should they escape rearing facilities. While environmental effects have been examined in some GH transgenic models, there is a lack of information on whether effects differ among different constructs or strains of transgenic fish. We compared growth and survival of wild-type coho salmon (*Oncorhynchus kisutch*) fry, a fast-growing GH transgenic strain containing a metallothionein promoter (T_MT_), and three lines/strains containing a reportedly weaker histone-3 promoter (T_H3_) in hatchery conditions and semi-natural stream tanks with varying levels of natural food and predators. Rank order of genotype size and survival differed with varying environmental conditions, both within and among experiments. Despite accelerated growth in hatchery conditions, T_MT_ fry gained little or no growth enhancement in stream conditions, had enhanced survival when food was limiting, and inconsistent survival under other conditions. Rank growth was inconsistent in T_H3_ strains, with one strain having highest, and two strains having the lowest growth in stream conditions, although all T_H3_ strains had consistently poor survival. These studies demonstrate the importance of determining risk estimates for each unique transgenic model independent of other models.

## Introduction

Insertion of a growth hormone (GH) transgene results in greatly accelerated growth in a number of commercially important finfish species (e.g. [1–5]), and has the potential to improve aquaculture production efficiency in some circumstances. Recently, an Atlantic salmon strain containing a chinook salmon GH transgene fused to an ocean pout antifreeze promoter (opAFP-GHc2) was approved for commercial production for the USA and Canada, potentially making it the first transgenic animal marketed for human consumption.

In addition to accelerated growth, GH transgenesis can result in pleiotropic effects for other traits, including altered foraging and predator avoidance behavior, disease resistance, reproductive potential, and swimming performance (see [6]). Consequently, concern exists regarding the potential environmental impacts GH transgenic fish may have on natural ecosystems, should they escape rearing facilities. The potential environmental risks of GH transgenic fish can be calculated as a factor of the potential for the fish to enter, persist, and disperse in the ecosystem (exposure) and the potential to adversely influence ecosystem components (harm, e.g. [6–8]). An important component in assessing environmental risk is determining the certainty with which exposure and harm can be predicted. The approval of GH transgenic Atlantic salmon for human consumption in the USA and Canada was based on physical and biological containment measures resulting in high certainty for negligible exposure of the transgenic fish to natural ecosystems [7, 9]. If economic and production incentives result in additional applications of novel GH transgenic fish for commercial production, these would require assessment for risk independent of the previously approved strains. However, available data for risk assessments may not be generated from the same strain, use the same gene construct, or be from the same species as that of a new application. This leads to the question to what degree can risk assessment-related data be applied with reasonable certainty among strains, constructs, or species of GH transgenic fish.

Research trials for GH transgenesis in commercially important fish species have utilized a variety of different gene constructs with different promoters and source species for transgene components (see [10]). Within-study comparisons have found that different lines (i.e. produced from the same founding individual but different F_1_ or later individuals, [11, 12]), strains (i.e. produced from different founding individuals using the same construct, [13, 14]), and promoter types [14] can result in different gains in growth rate within a species. In addition, a transgene construct that has been demonstrated to increase growth in one species (e.g. OnMTGH1 construct in coho salmon *Oncorhynchus kisutch*, [2]) may not produce accelerated growth in another species (e.g. OnMTGH1 construct in Nile tilapia, [4]). Whether observed differences among construct type, strains, or lines of transgenic fish in aquaculture environments would translate to similar differences in natural environments has not been examined. As data of GH transgenic fish is not available from natural environments, one option to estimate potential exposure or harm of different GH transgenic fish is to utilize semi-natural environments where conditions are kept close as possible to natural in a closed system. Sundström et al. [15] found GH transgenic coho salmon fry had greatly accelerated growth relative to wild type when reared in hatchery conditions, but had growth similar to that of wild type when reared in semi-natural stream conditions. As well, Leggatt et al. [16] found GH transgenic coho salmon fry grew faster than domesticated fry selected for fast growth in hatchery conditions, but that domesticated fry had greater growth and survival in semi-natural stream conditions. Such genotype-by-environment interactions make predicting relative success of different groups of fish from one environment to another (i.e. from hatchery conditions to natural environments) difficult to do with high certainty.

We compared the size and survival of coho salmon fry from wild-type and multiple GH transgenic strains in semi-natural conditions containing various levels of natural food items and/or predators, to determine if relative success of different types of GH transgenic fish is consistent under different semi-natural conditions. Strains compared were a well-studied GH transgenic coho salmon strain (M77, see [13]) containing a sockeye salmon GH1 transgene coupled to a metallothionein promoter from the same species (T_MT_, OnMTGH1), and three lines and strains of GH transgenic coho salmon containing the same GH1 transgene, but with a promoter reported to have lower growth promoting effects than the metallothionein promoter (histone 3 promoter from sockeye salmon, OnH3GH1, T_H3-A,B,C_, [14, 17]). This study has implications for determining uncertainty in risk assessments where extrapolation among transgenic constructs or strains is used.

## Materials and Methods

### Experimental fish

All experiments were conducted at Fisheries and Oceans Canada (DFO) Centre for Aquaculture and Environmental Research, West Vancouver, BC, Canada under institutional animal care permits meeting guidelines established by the Canadian Council for Animal Care. The experimental protocols were approved by the Pacific Region Animal Care Committee (Permit Numbers: 08–003, 10–017, 12–005). All handling of fish was performed under tricaine methanesulfonate anaesthesia buffered with sodium bicarbonate to minimize suffering. The facility used was specifically designed to prevent escape of GH transgenic fish to natural ecosystems. All fish were derived from the Chehalis River Hatchery strain, obtained from DFO’s Chehalis River Hatchery, Agassiz, BC, Canada. Several strains of transgenic fish (T) heterozygous for the sockeye salmon GH1 transgene were used. Promoters linked to the GH transgene were either a metallothionein (MT) promotor (OnMTGH1 transgene, [2]) or a histone 3 (H3) promotor (OnH3GH1 transgene, [14]), both from sockeye salmon. All OnMTGH1 fish (T_MT_) used were of the M77 strain produced from a single founding transgenic individual and used in the majority of previous studies examining GH transgenic coho salmon (e.g. [13]). Three strains/lines of OnH3GH1 (T_H3_) fish were used, produced from two founding G_0_ transgenic individuals previously reported to have moderate (H3-0474, or T_H3-A_) and low (H3-776E) growth rate respectively relative to MT strains [14]. The H3-776E strain was resurrected using cryopreserved milt from two males heterozygous for the OnH3GH1 transgene crossed with nine wild-type nature-reared females from the Chehalis River Hatchery. One of these H3-776E males produced expected slow-growing offspring relative to other T lines (T_H3-B_), while the other H3-776E male produced relatively fast-growing offspring (T_H3-C_). All transgenic lines were propagated by crossing males homozygous (T_MT_, T_H3-A_) or hemizygous (T_H3-B_, T_H3-C_) for the transgene with nature-reared wild-type females obtained from the Chehalis River Hatchery. T_MT_ and T_H3_ offspring were identified from their wild-type siblings by the presence of the MT or H3 GH transgene by PCR analysis (see [18], H3 specific forward primer 5’-3’: CCGACTGTGAAAGAAGGAAGCT). All wild-type (W) fish used were produced from crosses of nature-reared fish obtained from the Chehalis River Hatchery. The same maternal parents were used for both W and T fish produced in the same years. These H3 and MT (and CMV) promoter constructs (provided to Dr. H. Mölsä) were also found to cause strong growth elevation in founder transgenic Arctic char, but effects among constructs were not apparent in G_0_ fish [19].

**Fig 1:**
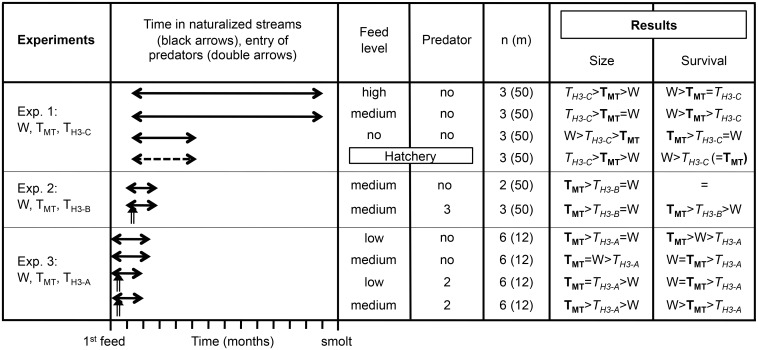
Experimental design and rank order results for assessing size and survival GH transgenic and wild genotypes. Genotypes are wild-type (W) and growth hormone transgenic strains including relatively high growth-stimulated OnMTGH1 (**T**_**MT**_) and OnH3GH1 (*T*_*H3-C*_) strains, a moderate growth-stimulated OnH3GH1 strain (*T*_*H3-A*_), and low growth-stimulated OnH3GH1 strain (*T*_*H3-B*_). Genotypes were reared together in semi-natural stream tanks landscaped with gravel (Exp. 1–2) or artificial turf (Exp. 3), rocks, logs, and artificial plants. Fish were fed natural food items 2 times (low), 5 times (medium) or 25 times (high) per week, or given no supplemental food. Fish were reared with no or 2–3 rainbow trout predatory smolts. n = number of tanks per experimental condition, and m = number of fish per genotype per tank. Experiment 1 included size and survival comparison in hatchery conditions with fish fed an artificial diet to satiation (dashed arrow). NOTE: in experiment 1, the ratio of W: T_H3-C_ was skewed so that approximately 55 W and 45 T_H3-C_ fish were included per tank.

Prior to experiments, fish were reared in hatchery conditions in aerated, flow-through well water (10 ± 1°C), with artificial daylight set to natural diurnal variation, and fed 2–6 times per day with a stage-appropriate commercial diet (Skretting Canada Ltd., Vancouver, BC, Canada). To estimate traits affecting fitness of fish groups in natural ecosystems, fish were reared in semi-natural, enclosed, stream tanks (see Fig 1 for summary of experimental design). Unless otherwise stated, stream tanks were long and shallow (4.3 × 0.9 × 0.4 m), with gravel bottoms and containing multiple refuge areas (hollow rocks, logs, artificial plants, see [15]). Stream tanks were covered with translucent acrylic sheeting, and lighting was natural, reduced in intensity by a white tent enclosure. Water supply was untreated, unfiltered creek water from nearby Cypress creek. Natural food items (e.g. caddis fly, stone fly and mayfly larvae) entered through the water supply. All supplemental food supplied to the fish in the stream tanks was natural (newly hatched brine shrimp, frozen blood worms and mysis shrimp, live juvenile crickets or fruit flies). Experimental hatchery conditions were as per pre-experimental hatchery conditions above. To ensure fish health, hatchery tanks were checked multiple times per day and the screened drain of each stream tank was checked daily for moribund fish or mortalities. If moribund fish were observed, these were removed and humanely euthanized by overdose of anaesthetic (100 g/L tricaine methanesulfonate anaesthesia buffered with 200 g/L sodium bicarbonate).

### Experiment 1: T_MT_ versus T_H3-C_ in hatchery conditions, or stream tanks with varying food levels

T_H3-C_ and W fish were produced by crossing seven heterozygous T_H3-C_ males with 19 wild-type females, resulting in approximately 55 W: 45 T_H3-C_ offspring. T_MT_ fish were taken from a stock population produced from 28 homozygous T_MT_ males crossed with 33 wild-type females (including those females used for W: T_H3-C_ crosses). Crosses were made Jan 25-Feb 9, and fish were ponded in May. Experiments were initiated June 9, where 50 T_MT_ and 100 mixed W plus T_H3-C_ were euthanized by an overdose of anaesthetic, mass and length measured, and W / T_H3-C_ fin-clipped for genotype ratio approximation. For hatchery growth, 50 T_MT_ and 100 mixed W plus T_H3-C_ fish (approximately 55 W: 45 T_H3-C_) were placed in each of three 200 L hatchery tanks supplied with well water and fed a size-appropriate standard commercial diet to satiation 2–6 times per day. For stream tank growth, 50 T_MT_ and 100 mixed W plus T_H3-C_ fish were place in each of 9 stream tanks supplied with creek water. Fish in three stream tanks were given one of: 1) no supplemental food beyond that entering in the natural water supply (no supplemental food), 2) natural food items once per day, 5 days per week, at random times per day, at random places within the stream tank, and at a level reported to produce typical growth rates in wild-type coho salmon fry reared in nature (medium supplemental food, [15]), or 3) natural food items 5 times per day, 5 days per week as above (high supplemental food). At 16 weeks, fish from all hatchery and stream tanks were removed, lightly anaesthetized (50 g tricaine methanesulfonate buffered with 100 g sodium bicarbonate), mass and length measured, and fin-clipped for genotype confirmation. At this point fish in the no supplemental food group were terminated due to low survival numbers, and fish in the hatchery group were terminated due to large size. Fish in the medium and high supplemental food groups were returned to the stream tanks and sampled as above at 51 weeks post stream tank entry. Fish were also sampled at 8 weeks (all groups) and 33 weeks (medium and high supplemental groups only). Results from these two times were similar to those of 16 and 51 weeks and are consequently not shown.

### Experiment 2: T_MT_ versus T_H3-B_ in streams tanks with or without predators

T_H3-B_ and W fish were produced by crossing one T_H3-B_ heterozygous male with 10 wild-type females, resulting in approximately 50 W: 50 T_H3-B_ offspring. T_MT_ fish were produced by crossing 10 T_MT_ homozygous males with the same 10 wild-type females used for the W / T_H3-B_ fish. Crosses were made Jan 17, and fish were ponded on March 18. On April 15, 100 mixed W plus T_H3-B_ fish and 50 MT fish were introduced to each of 5 stream tanks. The fish were fed a natural diet once per day, 5 times per week at random times and at one of three food points as per the medium supplemental food group in Experiment 1. At ten days, three rainbow trout predators (31.4 ± 1.1 g, 14.4 ± 0.2 cm) were introduced to three of the stream tanks and designated as + predator tanks (n = 3) while the remaining tanks were designated as–predator tanks (n = 2). Rainbow trout predators were trained to feed on fry prior to their introduction, and similar studies found this experimental design results in predation on and increased mortality of fry by predators [20, 21]. All fish were sampled (enumerated, mass, length, fin clipped for genotype confirmation) from each tank at 8 weeks after stream tank entry. Fish were also sampled at 4 weeks, and results from this time were similar to those of 8 weeks and are consequently not shown.

### Experiment 3: T_MT_ versus T_H3-A_ with varying food and predators

Eggs from five wild-type females were split into three groups and pair-crossed to five wild-type males (W fish), crossed to one homozygous T_H3-A_ male, or pair-crossed to five homozygous T_MT_ males. The study was conducted between June 4 and August 14. Initial average mass of fish was estimated by batch weighing a known number of each genotype. Twelve fish from each genotype were transferred to each of twenty-four 500 L (2.46 × 0.58 × 0.35 m) stream aquaria with 90% natural creek water and 10% well water, landscaped with natural rocks and artificial plants for refuge areas. Due to practical reasons gravel substrate could not be used, and artificial turf substrate was installed instead. Half the tanks were provided natural food items once a day 5 days a week at random times per day (medium supplemental food, as per Experiment 1) and the other tanks provided natural food items twice a week (low supplemental food). After 12 days, half the tanks received two hatchery-reared rainbow trout predators (17.8 ± 0.2 g, 11.6 ± 0.1 cm) that remained for 6 weeks resulting in a two-way design with factors being food level (low/medium) and predator (presence/absence). Two days after predators were removed, fry in these predator tanks were lightly anaesthetized, mass and length measured, and fin-clipped for genotype confirmation. Fry from non-predator tanks were weighed and measured after another 2 weeks.

### Statistical analyses

Statistical analyses were performed using R 3.2.1 [22]. Differences among initial genotype sizes were analyzed with the ANOVA function followed by Bonferroni *post-hoc* tests. Generalized linear mixed models were used to test for differences in variables within the experiments using the lme4 package [23]. In experiments with no more than one environmental condition (i.e. hatchery rearing) variables were analyzed with genotype as a fixed factor and genotype was crossed with the random factor tank [model: lmer (variable ~ Genotype + (1|Tank/Genotype)]. Where more than one environmental condition was present (i.e. different food levels, presence or absence of predator in stream tanks), variables were analyzed with genotype and environmental conditions as fixed interacting factors, and genotype was crossed with the random factor tank. Fish size was modelled using the normal distribution and identity link [model: lmer (variable ~ Genotype * Environmental Condition 1 * Environmental Condition 2 + (1|Stream/Genotype)], and mass data was ln-transformed prior to analyses to meet equal variance assumptions. Survival was modelled using a binomial distribution and log-link function, adding the individual observations (i.e. tank) as a random factor to account for overdispersion: [glmer (survival ~ Genotype * Environmental Condition 1 * Environmental Condition 2 + (1|Stream/Genotype) + (1/obs)]. Significance of the model outputs were evaluated using the Anova() function in the car package [24] with test type 3, and post-hoc comparisons were performed using model-predicted values (predict() function in lme4 package) and paired t-tests. All data are presented as means ± standard error of the mean. Supporting datasets are given in SI Dataset.

## Results

### Experiment 1: T_MT_ versus T_H3-C_ in hatchery conditions, or stream tanks with varying food levels

**Fig 2:**
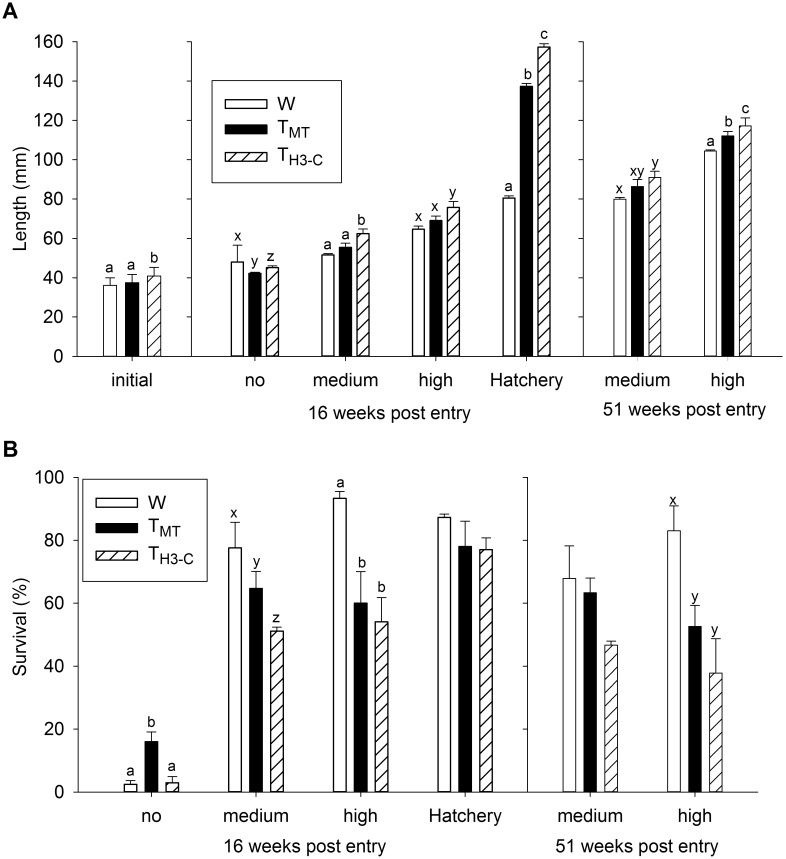
Comparison of genotypes under hatchery conditions or in semi-natural streams with varying food levels. Experiment 1. A) Length and B) survival of wild-type (W) and growth hormone transgenic strains (T_MT_ and T_H3-C_) of coho salmon fry reared in semi-natural stream conditions with no, medium, or high levels of supplemental natural food items, or in hatchery conditions for 16 weeks (all) and 51 weeks (medium and high supplemental food only). Initial length at the start of the experiment is also included. n = 3 tanks per treatment, with approximately 50 individuals per genotype per tank. Letters indicate significant differences among genotypes within food level/environmental condition and time (*P* < 0.05), and data is given as mean ± s.e.m.

Size (mass and length) and survival of W, T_MT_, and T_H3-C_ fish were compared in stream tanks under no (experiment run for 16 weeks), and medium and high (examined both after 16 and 51 weeks) supplemental food levels, as well as in standard hatchery conditions (run for 16 weeks). T_H3-C_ fish began the experiment 1.4-fold larger in mass (F_2,149_ = 13.4, *P* < 0.001, Table 1) and 1.1-fold longer in length (F_2,149_ = 18.3, *P* < 0.001, Fig 2A) than T_MT_ and W fish. At 16 weeks, genotype rank order in mass (Table 1) and length (Fig 2A) were dependent on food level (see Table 2 for all factor and interaction statistics). In the most limiting conditions (no supplemental food) W fry were greater in size (mass and length) than T fry, and T_H3-C_ fry were greater in size than T_MT_ fry. In less limiting stream (medium and high food) and hatchery conditions, T_H3-C_ were larger than T_MT_ or W fry, and T_MT_ were only able to gain size advantage over W fry in hatchery conditions. At 51 weeks when fed medium supplemental food, T_H3-C_ fish were greater in size than W fish only, while at high supplemental food, size was in the order of T_H3-C_ > T_MT_ > W (see Table 1, Fig 2A).

**Table 1:** Experiment 1 mass (g) of wild-type and GH transgenic fry in hatchery or stream tanks with varying food levels.

Time	Conditions–Food level	W	T_MT_	T_H3-C_
Initial		0.51 ± 0.20^x^	0.58 ± 0.22^x^	0.74 ± 0.25^y^
16 wk	Stream–no	1.18 ± 0.42^x^	0.76 ± 0.05^y^	1.12 ± 0.28^x^
16 wk	Stream–medium	1.76 ± 0.16^x^	2.20 ± 0.40^xy^	3.01 ± 0.42^y^
16 wk	Stream–high	3.94 ± 0.35^a^	4.91 ± 0.49^ab^	6.14 ± 0.62^b^
16 wk	Hatchery	6.35 ± 0.57^x^	34.44 ± 0.82^y^	46.47 ± 1.72^z^
51 wk	Stream–medium	6.62 ± 0.21^a^	8.24 ± 0.77^ab^	9.42 ± 1.03^b^
51 wk	Stream–high	14.85 ± 0.28^x^	19.38 ± 1.31^y^	20.32 ± 1.82^z^

Fish groups are wild-type (W) and two strains of GH transgenic coho salmon fry (T_MT_ and T_H3-C_). Groups were sampled initially, then reared together in standard hatchery conditions with artificial food, or in naturalized stream tanks with no, medium, or high supplemental natural food provided. Fish were sampled after 16 weeks (all groups) and 51 weeks (medium and high food levels only). Letters indicate significant differences among genotypes within environmental conditions and time (*P* < 0.05).

**Table 2:** χ^2^ values and *P* values, presented as χ^2^ (*P*), for interaction and factor effects for experiments conducted, where *P* < 0.05 is considered significant.

Experiment	Variable	F x P x G	F x G	P x G	F x P	G	F	P
Exp. 1 (16 wk)	mass	n/a	290.0 (<0.001)	n/a	n/a	*1*.*6 (0*.*448)*	*506*.*4 (<0*.*001)*	n/a
Exp. 1 (16 wk)	length	n/a	954.6 (<0.001)	n/a	n/a	*0*.*8 (0*.*683)*	*1613 (<0*.*001)*	n/a
Exp. 1 (16 wk)	survival	n/a	36.5 (<0.001)	n/a	n/a	*3*.*7 (0*.*158)*	*61*.*8 (<0*.*001)*	n/a
Exp. 1 (51 wk)	mass	n/a	0.1 (0.952)	n/a	n/a	17.8 (<0.001)	61.6 (<0.001)	n/a
Exp. 1 (51 wk)	length	n/a	1.1 (0.586)	n/a	n/a	18.0 (<0.001)	62.0 (<0.001)	n/a
Exp. 1 (51 wk)	survival	n/a	6.2 (0.044)	n/a	n/a	*5*.*4 (0*.*067)*	*0*.*8 (0*.*362)*	n/a
Exp. 2	mass	n/a	n/a	3.9 (0.141)	n/a	21.7 (<0.001)	n/a	9.4 (0.002)
Exp. 2	length	n/a	n/a	4.6 (0.098)	n/a	21.0 (<0.001)	n/a	10.2 (<0.001)
Exp. 2	survival	n/a	n/a	10.4 (0.006)	n/a	*4*.*9 (0*.*087)*	n/a	*81*.*4 (<0*.*001)*
Exp. 3	mass	0.4 (0.810)	9.6 (0.008)	5.7 (0.059)	0.0 (0.843)	*29*.*4 (<0*.*001)*	*4*.*0 (0*.*047)*	4.9 (0.027)
Exp. 3	length	2.2 (0.331)	14.5 (0.001)	6.0 (0.050)	1.0 (0.321)	*35*.*8 (<0*.*001)*	*0*.*5 (0*.*465)*	5.1 (0.024)
Exp. 3	survival	0.8 (0.677)	7.1 (0.028)	0.5 (0.762)	0.2 (0.666)	*20*.*5 (<0*.*001)*	*1*.*3 (0*.*264)*	26.3 (<0.001)

Factors are Food Level (F), Predator Presence (P), and Genotype (G), and n/a indicates factor/interaction was not included in specific experiment. Where interactions are significant, individual factor effects are given in italicized text to indicate effects should be examined within other factor effects.

At 16 weeks genotype rank order in survival was dependent on food level (Fig 2B, Table 2). In the most limiting conditions (no supplemental food), T_MT_ had 5.9-fold greater survival than T_H3-C_ and W fish, while in less limiting conditions W fry generally had the highest survival. At medium supplemental food, survival was in the order of W > T_MT_ > T_H3-C_, while at high supplemental food survival was in the order W > T_MT_ = T_H3-C_. Genotypes did not significantly differ in survival after hatchery rearing. At 51 weeks, there a significant interaction between genotype and food level, where at high supplemental food only, W fry had 1.7-fold greater survival than either T group (Fig 2B, Table 2).

### Experiment 2: T_MT_ versus T_H3-B_ in streams tanks with or without predators

**Fig 3:**
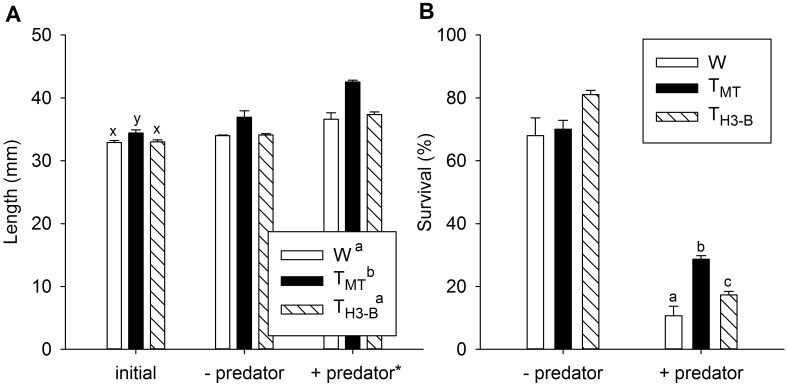
Comparison of genotypes in semi-natural streams with or without predators. Experiment 2. A) Length and B) survival of wild-type (W) and growth hormone transgenic strains (T_MT_ and T_H3-B_) of coho salmon fry reared in semi-natural stream conditions under medium supplemental food level, without (-) or with (+) two rainbow trout predatory smolts, measured at the initiation of the experiment (length only), and after 8 weeks. Predatory smolts were introduced at 10 days. n = 2 stream tanks for -predator and n = 3 stream tanks for +predator treatments, with 50 individuals per genotype per tank * indicates factor effect of predator presence, letters on legend indicates factor effect of genotype, letters on bars indicate significant effect of genotype within environmental condition (*P* < 0.05), and data is given as mean ± s.e.m.

Size and survival of W, T_MT_, and T_H3-B_ fish were compared in stream tanks under medium supplemental food levels with or without predators (8 weeks). Prior to entering the stream tanks, T_MT_ fish were 1.2-fold greater in mass than W but not T_H3-B_ fish (F_2,143_ = 3.5, *P* = 0.034, Table 3) and 1.04-fold greater in length than both T_H3-B_ and W fish (F_2,143_ = 4.3, *P* = 0.015, Fig 3). After 8 weeks, T_MT_ had a size advantage over T_H3-B_ and W fish in both mass (1.5-fold, Table 3) and length (1.1-fold, Fig 3, see Table 2 for all factor and interaction statistics), regardless of predator presence, while T_H3-B_ and W fry did not differ in size. Genotype rank order survival depended on presence of predators in the stream tanks (Fig 3). In the absence of predators, there was no significant difference in survival among genotypes, while in the presence of predators survival rank order was T_MT_ > T_H3-B_ > W. All genotypes had greater size and lower survival in the presence of predators than in their absence.

**Table 3:** Experiment 2 mass (g) of wild-type and GH transgenic fry in stream tanks with medium food levels and or without predators.

Stream conditions	W	T_MT_	T_H3-B_
Initial	0.30 ± 0.01^x^	0.36 ± 0.02^y^	0.31 ± 0.01^xy^
No predators	0.33 ± 0.01^a^	0.49 ± 0.02^b^	0.34 ± 0.01^a^
With predators	0.39 ± 0.04^x^	0.71 ± 0.02^y^	0.45 ± 0.00^x^

Fish groups are wild-type (W) and two strains of GH transgenic coho salmon fry (T_MT_ and T_H3-B_). Groups were sampled initially, then reared together for 8 weeks in naturalized stream tanks with medium supplemental natural food provided and with or without three rainbow trout smolt predators. Letters indicate significant differences among genotypes within predator condition (*P* < 0.05).

### Experiment 3: T_MT_ versus T_H3-A_ with varying food and predators

**Fig 4:**
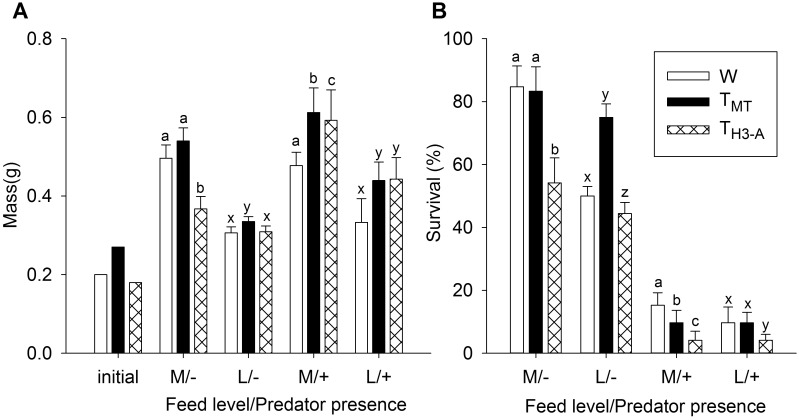
Comparison of genotypes in semi-natural aquaria with varying food and predator levels. Experiment 3. A) Mass and B) survival of wild-type (W) and growth hormone transgenic strains (T_MT_ and T_H3-A_) of coho salmon fry reared in semi-natural aquaria with medium (M) or low (L) feeding levels, with (+) or without (-) two rainbow trout smolt predators. Predators were introduced at 12 days, and variables given at initiation of the experiment (batch mass) and at 8 weeks (+ predator aquaria) or 10 weeks (- predator aquaria) rearing in the stream aquaria. n = 6 aquaria per treatment, with 12 individuals per genotype per tank. Initial mass of groups was estimated by batch weighing a known number fish in each genotype. Letters indicate significant differences among genotypes within environmental condition (*P* < 0.05), and data is given as mean ± s.e.m.

Size (mass and length) and survival of W, T_MT_, and T_H3-A_ fish were compared in stream aquaria under low or medium supplemental food levels, and with (8 weeks) or without (10 weeks) predators. At the start of the experiment, batch measuring of fry estimated initial mass to be 0.27, 0.18 and 0.20 g for T_MT_, T_H3-A_, and W fish respectively. When genotypes were compared within food level/predator presence, genotype ranking varied depending on the environmental conditions (see Table 2 for all interaction and factor statistics). Mass rank orders (Fig 4A) were T_MT_ = W > T_H3-A_ in medium food conditions without predators, T_MT_ > T_H3-A_ > W in medium food in the presence of predators, T_MT_ > T_H3-A_ = W in low food without predators, and T_MT_ = T_H3-A_ > W in low food in the presence of predators. Trends in length were similar to mass, but with fewer significant differences (Table 4). When genotype survival was compared within food level/presence of predator, T_H3-A_ had lower survival than the other two genotypes regardless of condition (see Fig 4B). In medium food and no predators, as well as low food and predators, T_MT_ and W fish had similar survival, while T_MT_ fish had higher survival with low food and no predators and W fish had higher survival with medium food and in the presence of predators.

**Table 4:** Experiment 3 length (mm) of wild-type fish and GH transgenic fry reared in stream aquaria with varying food and predator load.

Food level/Predator	W	T_MT_	T_H3-A_
Medium/No	37.2 ± 0.4^a^	38.5 ± 0.4^a^	34.9 ± 0.4^b^
Medium/Yes	36.5 ± 0.1^x^	39.4 ± 0.2^y^	38.7 ± 0.1^y^
Low/No	34.0 ± 0.1^a^	34.6 ± 0.1^b^	34.3 ± 0.1^ab^
Low/Yes	35.0 ± 0.1^a^	36.2 ± 0.1^b^	35.7 ± 0.1^ab^

Fish groups are wild-type (W) and two strains of GH transgenic coho salmon fry (T_MT_ and T_H3-A_). Groups were reared together in naturalized stream aquaria with low or medium supplemental natural food provided, and with or without two rainbow trout predatory smolts. Fish were sampled after 8 weeks (predator aquaria) or 10 weeks (no predator aquaria). Letters indicate significant differences among genotypes within environmental condition (*P* < 0.05).

## Discussion

The majority of data relevant to risk assessments for aquaculture-related GH transgenic species have been generated in only a few models (OnMTGH1 coho salmon and rainbow trout, opAFPGHc2 Atlantic salmon, pCAgcGH common carp, e.g. [15, 25–27]). It would be expeditious to use data from well-studied models when estimating risks of novel GH transgenic fish, but the reliability of this approach has not been fully assessed. Genotype × environment (G × E) interactions have been observed for many fitness components when comparing wild-type and T_MT_ coho salmon (see [6]) and G × E interactions have been observed when comparing different types of fast-growing salmonids (i.e. GH transgenic versus domesticated fry [16, 27]). To examine whether transgene construct or strain may influence risk assessment-related success of GH transgenic animals, we measured the growth and survival of a well-studied GH transgenic coho salmon strain (T_MT_) containing a GH construct with a metallothionein promoter with several strains of GH transgenic fish containing a histone 3 (H3) promoter (T_H3-A,B,C_ strains) under semi-natural conditions mimicking varying conditions found in nature (i.e. varying levels of natural food and predators). In particular we examined whether rank order of size and survival of different genotypes would be altered by environmental conditions to determine if different GH transgenic lines respond to different environmental variables equally (i.e. are G × E interactions present among transgenic strains). In the current study the relative rank order of survival and size for W, T_MT_ and T_H3_ lines was altered by environmental conditions within and among experiments (see Fig 1 for summary of rank results), demonstrating G × E interactions exist among different GH transgenic models, both among and within construct types. As such, extrapolating risk estimates among strains of GH transgenic fish cannot be done with a high degree of certainty.

### Influence of transgene promoter and strain on relative size in different environments

Size has been well established to influence ecologically relevant phenotypes such as dominance hierarchies and foraging competition (e.g. [28]). The T_MT_ strain containing the OnMTGH1 construct has been used for all simulated stream studies examining ecological effects of GH transgenic coho salmon (e.g. [15]), and was included in all current experiments. The relative size of T_MT_ and W fry under similar conditions in different experiments was fairly consistent. While the T_MT_ strain of GH coho salmon obtained greatly increased size over W fry in hatchery conditions in the current and previous studies (e.g. [13, 15]), in stream tanks, T_MT_ trended towards modest size increase above that of W fish where supplemental food was given, regardless of predator presence, although this difference was not significant in all experiments. Greatly increasing natural supplemental food items in streams did not bring T_MT_ size up to that of hatchery-reared fish, although removing supplemental food resulted in smaller size relative to W fish despite larger initial size in T_MT_ fish (Experiment 1). This concurs with previous studies reporting modest or no increase in growth in T_MT_ fish under semi-natural stream conditions and natural food supply [15, 16, 20, 21], and indicates T_MT_ fry would not gain size advantage over wild fish in most natural conditions, and potentially be at a size disadvantage in severely limiting conditions, despite intrinsic potential for accelerated growth. As well, Sundt-Hansen et al. [29] found exogenous GH increased growth of Atlantic salmon fry in hatchery conditions, but had a negative effect on growth in a natural stream, indicating excessive GH may have underlying negative consequences in natural ecosystems at this lifestage.

In contrast to T_MT_ fish, the rank order of size of T_H3_ fry varied with environmental conditions, and the three T_H3_ lines used had different relative size in equal conditions among experiments. T_H3_ strains were previously reported to have slower growth than T_MT_ strains in hatchery conditions [14], and the present study sought to determine whether this trend would continue in semi-natural conditions. T_H3-B_ fry under medium food levels with or without predation and T_H3-A_ fry under medium or low food levels without predation had expected lower size than T_MT_ fry, and equal or lower size than W fish, indicating these two strains would be unable to obtain a size advantage outside hatchery conditions in most circumstances, although T_H3-A_ obtained larger size than W fish under predation. In contrast, fish from the T_H3-C_ line were initially larger than T_MT_ and W fish, and maintained this larger size in all conditions except the most limiting (no supplemental food), indicating they have the potential to obtain size advantage in many natural conditions. This is surprising given the previous slow growth of this strain [14], and the slow growth of its sister line (T_H3-B_). T_H3-C_ and T_H3-B_ lines were resurrected from cryopreserved milt of two males produced from the same founding line. The higher growth of the T_H3-C_ line may indicate that a structural change has occurred with this transgene that confers higher GH expressions and elevated growth relative to what has been observed in earlier lines of this strain. This phenomenon has also been observed in GH transgenic carp and trout, where two lines of transgenic fish produced from the same founding line resulted in different inheritable growth [11, 12]. When the three T_H3_ lines were compared under similar conditions in the three experiments (medium food level, no predation), the three strains showed very different rank order in size (i.e. T_H3-C_ had greatest size in Exp. 1; T_H3-B_ had equal size to W and smaller size than T_MT_ in Exp. 2; and T_H3-C_ were the smallest in Exp. 3). This demonstrates that different lines with equal transgene constructs may be at a size advantage, disadvantage, or neither, in similar conditions, although differences among experiments may also have been influence by time and size of entry into stream tanks, as has been observed in T_MT_ fish [30].

### Influence of transgene promoter and strain on relative survival in different rearing environments

Survival is a defining component of fitness, and survival relative to wild fry indicates the potential for a strain to persist in an ecosystem. Unlike relative size, relative survival of T_MT_ versus W fry varied under different conditions within experiments, as well as under similar conditions across experiments, indicating the success of T_MT_ fish is highly dependent on specific environmental conditions. Under limiting conditions (low or no supplemental food), T_MT_ gained a survival advantage over W fish (Exp. 1 and 3), while in less limiting conditions T_MT_ had either lower (medium and high supplemental food, Exp. 1) or equal survival (medium supplemental food, Exp. 2, 3) to W fry, demonstrating survival advantage of T_MT_ fish can be removed or reversed with different food availability. Other studies have also demonstrated a survival advantage of T_MT_ over W fish in severe growth-limiting conditions [20, 21]. Due to low anti-predator behavior observed in T_MT_ strain fish [31, 32], GH transgenic fish are expected to have lower survival than W fish under predation pressure, as has been observed in some studies [20, 33], but not others [21]. T_MT_ fry had inconsistent relative survival in the presence of predators, where survival was higher than W fry at medium food levels in Exp. 2 but lower in Exp. 3, and equal under low food levels in Exp. 3. Difference in rank survival of T_MT_ and W fry within experiments may be due to increased foraging and decreased predator avoidance behavior observed in GH transgenic fish [27, 31, 32, 34–36], providing advantages or disadvantages to T_MT_ fry depending on food availability and predator presence. The factors causing differences in rank order among equal conditions in different studies are not known, but could include age and relative size at entry, length of trial, competitive ability of different co-habiting T_H3_ strains, and/or amount of refuge areas in substrate (i.e. as provided by gravel versus artificial turf in Experiment 2 versus 3 respectively), all which may influence the potential to form dominance hierarchies for shelter spaces, or influence cannibalistic or risk taking behavior in T_MT_ and/or W fry (e.g. [27, 32, 37, 38]).

Although T_H3-C_ and T_MT_ fry had similar survival in the richest conditions (high food level in semi-natural streams, and hatchery conditions), in all other conditions examined T_H3_ strains had lower survival than T_MT_ fry, and T_H3_ strains had lower survival than W fry in all conditions except T_H3-C_ under no supplemental food and T_H3-B_ under medium food level with or without predation. There appears to be a greater survival cost to T_H3_ strains under limiting conditions relative to T_MT_, regardless of whether they have lower (T_H3-B_ and T_H3-A_) or higher (T_H3-C_) growth than T_MT_, suggesting this genotype may have limited capacity to persist in natural ecosystems. The mechanisms driving the lower survival of GH transgenic fish containing the H3 promoter are not known as physiological and behavioral pleiotropic effects have not been examined for this construct, but may indicate detrimental environmental regulation of the H3 transgene promoter in stream tank conditions. Future experiments examining foraging, competitive, and predator avoidance behaviours could determine underlying causes of different growth and survival of GH transgenic strains in semi-natural environments, and aid in understanding potential for different transgenic strains to persist in natural environments.

## Conclusions

This study demonstrates the potential for GH transgenic coho salmon fry to persist or gain a size advantage in natural ecosystems can be strongly influenced by transgenic strain, as well as G x E interactions. This concurs with previous experiments that found strong G x E when comparing GH transgenic and domesticated salmonid fry [16, 27], and indicates such interactions may be a common when comparing performance of different model organisms with altered intrinsic growth. In all experiments T_H3_ strains responded to shifts in environmental conditions differently than T_MT_ fry, demonstrating risk estimates cannot be extrapolated with certainty among different strains and promoters in GH transgenic coho salmon. T_MT_ had higher or similar survival and greater growth than wild-type in most examined scenarios, indicating that, as fry, this strain has potential to persist in and impact some natural environments with ecosystem components similar to those examined here. Low survival in all T_H3_ GH transgenic strains indicates limited ability to persist in natural ecosystems, regardless of whether they were able to gain a size advantage over wild-type fish, although the larger size of the T_H3-C_ strain suggests potential to impact wild fry populations through dominance hierarchies and competition at this stage. However, overall T_H3_ strains of GH transgenic fry may have lower risk estimates than T_MT_ fry, at least in conditions similar to those examined here. This study demonstrates the remarkable plasticity of response of different genotypes to variable environmental conditions, and the uncertainty inherent in predicting risk by extrapolating data among different environments and from different strains and species of GH transgenic fish.

## Supporting Information

S1 Dataset Raw datasets generated from Experiments 1–3. (XLSX)
